# Validity and Sensitivity of Extended Field‐of‐View Wireless Palm‐Sized Ultrasonography for Assessing Muscle Hypertrophy

**DOI:** 10.1002/ejsc.70234

**Published:** 2026-07-30

**Authors:** Tatsuki Fukumitsu, Yuto Hashimoto, Sari Kanbayashi, Madoka Ogawa, Mizuki Sudo, Takanobu Okamoto, Soichi Ando

**Affiliations:** ^1^ Graduate School of Informatics and Engineering The University of Electro‐Communications Tokyo Japan; ^2^ Faculty of Sport Science Nippon Sports Science University Tokyo Japan; ^3^ Meiji Yasuda Health Promotion Center Meiji Yasuda Health Development Foundation Tokyo Japan; ^4^ Institute for Liberal Arts Institute of Science Tokyo Tokyo Japan; ^5^ Physical Fitness Research Institute Meiji Yasuda Life Foundation of Health and Welfare Tokyo Japan

**Keywords:** magnetic resonance imaging, muscle hypertrophy, reliability, skeletal muscle, ultrasonography

## Abstract

This study evaluated the validity and sensitivity of an extended field‐of‐view (EFOV) wireless palm‐sized ultrasound device for assessing quadriceps femoris cross‐sectional area (CSA), using magnetic resonance imaging (MRI) as the reference. Although previous studies have examined EFOV ultrasound, data validating EFOV wireless palm‐sized devices against MRI using large cohorts and training interventions have not been reported. In Experiment 1, midthigh CSA was measured in 100 young adults (75 males, 25 females) using ultrasound and MRI. Intra‐rater reliability was assessed via intraclass correlation coefficients (ICC), standard error of measurement (SEM), coefficient of variation (CV), and smallest detectable change (SDC). In Experiment 2, 11 males underwent an 8‐week resistance training program. Pre‐to‐post changes in CSA (ΔCSA) were compared between the two imaging modalities. In Experiment 1, the device demonstrated excellent reliability for males (ICC = 0.996; SEM = 0.85 cm^2^; CV = 1.19%; SDC = 2.35 cm^2^) and females (ICC = 0.993; SEM = 0.95 cm^2^; CV = 1.73%; SDC = 2.63 cm^2^). A strong correlation (*r* = 0.98, *p* < 0.001) was observed between ultrasound and MRI, with a mean bias of −1.23 ± 2.70 cm^2^. In Experiment 2, both modalities detected significant hypertrophy (*p* < 0.001). The mean ΔCSA measured by ultrasound (3.77 cm^2^, 5.98%) exceeded the SDC (2.35 cm^2^). ΔCSA correlated strongly between modalities (*r* = 0.92, *p* < 0.001). The EFOV wireless palm‐sized ultrasound device is a valid and sensitive tool for quantifying quadriceps femoris CSA and monitoring muscle adaptations, suitable for athletic performance and clinical settings.

## Highlight

- We evaluated the validity and sensitivity of a novel extended field‐of‐view (EFOV) wireless palm‐sized ultrasound device for quantifying quadriceps femoris cross‐sectional area (CSA).
- The device showed excellent intra‐ and inter‐rater reliability for both sexes, exhibiting a very strong correlation with gold‐standard MRI measurements.
- A longitudinal resistance training intervention confirmed that the EFOV wireless palm‐sized device is highly sensitive in detecting modest, exercise‐induced muscle hypertrophy.
- This wireless, compact imaging system provides a robust, point‐of‐care tool for real‐time muscle CSA tracking in both athletic performance and clinical rehabilitation settings.

**Abbreviations**
B‐mode: Brightness mode
CSA: Cross‐sectional area
CT: Computed tomography
CV: Coefficient of variation
EFOV: Extended field‐of‐view
ICC: Intraclass correlation coefficient
MRI: Magnetic resonance imaging
RM: Repetition maximum
RT: Resistance training
SDC: Smallest detectable change
SEM: Standard error of measurement

## 1 Introduction

Human skeletal muscle exhibits remarkable plasticity, undergoing structural and functional adaptations in response to resistance training (RT), aging, and various pathological conditions (Furrer et al. 2023). Accurate quantification of skeletal muscle mass is therefore essential not only for evaluating exercise‐induced adaptations but also for the clinical diagnosis of sarcopenia, cachexia, and metabolic disorders. Although several methodologies exist for muscle mass assessment, ultrasonography has emerged as a versatile and reliable tool (Franchi et al. 2018; Nijholt et al. 2017). Its primary advantage lies in its noninvasive nature, which facilitates the longitudinal monitoring of muscle mass changes during strength training or rehabilitative interventions.

To overcome the restricted field‐of‐view of standard Brightness mode (B‐mode) ultrasonography, muscle cross‐sectional area (CSA) is frequently assessed using the extended field‐of‐view (EFOV) technique. By continuously sweeping the ultrasound probe across the skin, EFOV generates a panoramic image of the entire muscle (Ahtiainen et al. 2010; Franchi et al. 2020; Hernandez‐Belmonte et al. 2022; Kunimasa et al. 2026; Minnehan et al. 2023; Scott et al. 2017; Stokes et al. 2021; Van den Broeck et al. 2023). This panoramic reconstruction relies on texture mapping algorithms that merge sequential images collected during real‐time scanning into a single large composite image (Franchi et al. 2018). Importantly, the accuracy of free‐hand CSA measurement via EFOV can be operator‐dependent (Hernandez‐Belmonte et al. 2022). Potential sources of user error include probe alignment, the pressure applied, the movement velocity, or the deviations from the target region during the image acquisition (Franchi et al. 2018; Hernandez‐Belmonte et al. 2022). To minimize these errors and ensure high reliability, a certain level of technical skill and familiarization with the device is required.

Recent evidence has shown that a novel wireless palm‐sized ultrasound device equipped with the EFOV technique can accurately assess muscle CSA in middle‐aged and older adults (Matsui et al. 2024). This study demonstrated high intra‐rater (intraclass correlation coefficient [ICC] > 0.99) and inter‐rater (ICC: 0.993) reliabilities as well as strong correlation with computed tomography (CT) measurements in the supine (*r* = 0.949) and seated (*r* = 0.958) positions (Matsui et al. 2024). This device is distinctive in that it features EFOV capability despite its compact size, and is uniquely optimized for CSA quantification rather than conventional diagnostic imaging. However, a previous study was limited to older populations with smaller muscle dimensions. Since younger adults typically exhibit larger CSAs and greater hypertrophy potential (Straight et al. 2020), validating this device across a broader morphological range, including individuals with higher muscle mass, and during dynamic muscle growth, is essential to ensure its utility in athletic and rehabilitative contexts. Furthermore, it is crucial to investigate the extent to which this EFOV wireless palm‐sized device can detect exercise‐induced muscle hypertrophy. Additionally, although CT is widely utilized in clinical research for its ability to distinguish lean tissue from adipose tissue via x‐ray attenuation (Goodpaster et al. 2000), magnetic resonance imaging (MRI) remains the gold standard for the precise segmentation of skeletal muscle (Pons et al. 2018; Stokes et al. 2021). Therefore, validating this novel device against MRI is highly warranted.

To this end, the primary objective of this study was to evaluate the validity of the EFOV wireless palm‐sized ultrasound device for assessing muscle CSA in young adults, using MRI as the reference criterion. The secondary objective was to determine the device's sensitivity in detecting longitudinal changes in muscle mass following an RT intervention. This investigation provides critical insights into the practical application of wireless, compact imaging for muscle CSA quantification in both athletic and clinical settings.

## 2 Materials and Methods

### 2.1 Ethics

All participants provided written informed consent prior to the commencement of the study. The protocols for both Experiment 1 and Experiment 2 were approved by the Institutional Review Boards of the University of Electro‐Communications (approval no. 20016) and the Nippon Sport Science University (approval no. 023‐H188). All procedures were conducted in accordance with the ethical standards of the latest revision of the Declaration of Helsinki, with the exception of registration in a public clinical trial database.

### 2.2 Experiment 1: Participants

A total of 100 participants were enrolled in Experiment 1. Their physical characteristics are summarized in Table 1. According to the Participant Classification Framework (McKay et al. 2022), the cohort comprised individuals with diverse levels of physical activity, ranging from trained/developmental individuals (Tier 2) who engaged in regular RT (≥ 3 times per week with an intention to improve strength) to sedentary individuals (Tier 0) who failed to meet the minimum physical activity guidelines (< 150 min/week of moderate‐intensity activity). This diversity was intended to ensure a broad morphological range of muscle dimensions within the sample. None of the participants reported a history of musculoskeletal injuries affecting the quadriceps femoris muscles.

**TABLE 1:** Physical characteristics of the participants.

	All (*n* = 100)	Male (*n* = 75)	Female (*n* = 25)
Age, years	23.0 ± 3.7	23.0 ± 4.0	22.7 ± 2.3
Height, m	1.68 ± 0.08	1.71 ± 0.06	1.58 ± 0.05
Body mass, kg	61.1 ± 9.7	63.3 ± 9.5	54.5 ± 6.7
Body mass Index, kg/m^2^	21.6 ± 2.7	21.5 ± 2.7	21.6 ± 2.6
Body fat, %	18.1 ± 6.5	15.7 ± 5.0	25.3 ± 5.2

### 2.3 Experiment 1: Procedures and Measurement

Body mass and body fat percentage were assessed via bioelectrical impedance analysis (InBody Co. Ltd., Seoul, South Korea). Both ultrasound and MRI assessments were performed on the same day. To evaluate the CSA of the quadriceps femoris, transverse images were acquired at 50% of the distance from the greater trochanter to the lateral femoral condyle (Ogawa et al. 2023), with the imaging plane oriented perpendicular to the long axis of the femur. This specific location was chosen because previous studies have demonstrated the feasibility and high accuracy of measurements at this site (Matsui et al. 2024; Noorkoiv et al. 2010).

To minimize potential operator‐dependent errors and ensure high reliability, all measurements were performed by two investigators who had extensive experience in musculoskeletal ultrasound and completed a designated pilot training period prior to data collection. Images were captured using an EFOV wireless palm‐sized ultrasound device (UT‐2000, Furuno Electric Co. Ltd., Hyogo, Japan; dimensions: 96 × 65 × 63 mm [width × length × height]; weight: approximately 260 g). The battery is rated to last approximately 7 hours under intermittent operation (2 min of scanning followed by a 3‐min interval).

**FIGURE 1:**
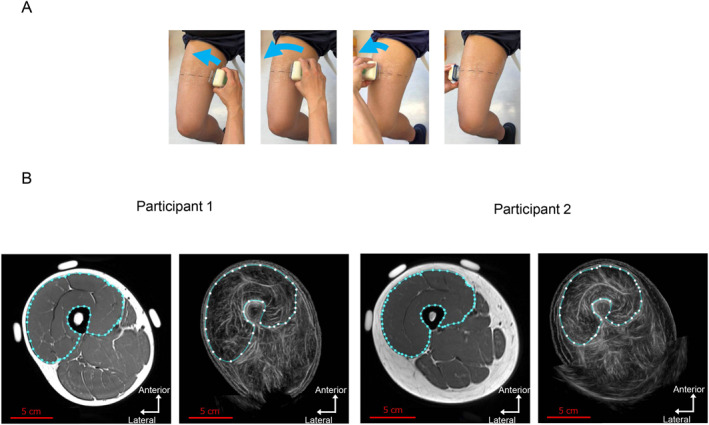
Measurement of the quadriceps femoris muscle cross‐sectional area (CSA). (A) Experimental setup demonstrating the use of the extended field‐of‐view wireless palm‐sized ultrasound device. (B) Comparison of representative CSA images from magnetic resonance imaging (left) and ultrasonography (right) from two participants. Participant 1 was a male (age: 23 years; height: 1.79 m; body mass: 67.3 kg), and Participant 2 was a female (age: 23 years; height: 1.59 m; body mass: 54.2 kg).

Operator 1 measured 75 male participants, and operator 2 measured 25 female participants. Images were acquired in the axial plane using a sector‐scan probe with a center frequency of 3 MHz. The field‐of‐view (FOV) was set to a 90° sector angle with an imaging depth of 160 mm. Participants were seated with their knees flexed at 90°, a posture previously shown to yield higher accuracy than supine measurements with this device (Matsui et al. 2024). To avoid muscle compression, the probe was applied with minimal, consistent pressure and ample ultrasound transmission gel to ensure optimal acoustic coupling. The device generates panoramic B‐mode images via a unidirectional sweep along a predefined skin marker (Figure 1A). Specifically, multiple narrow field‐of‐view images were acquired and reconstructed into a single panoramic image, which was then transmitted to a Microsoft Surface Pro tablet via a wireless Wi‐Fi connection to allow for review within approximately 15 seconds. Although the system automatically detected initial muscle boundaries, all segmentations were manually verified and adjusted by the operators (Figure 1B).

For the MRI measurements, participants were positioned supine, and the CSA was assessed using a 1.5 T whole‐body MRI scanner (Echelon Oval, Hitachi Medical Systems, Tokyo, Japan). A T1‐weighted spin‐echo sequence was employed with the following parameters: repetition time, 520 ms; echo time, 8.0 ms; slice thickness, 5 mm; and interslice gap, 0 mm. Images were reconstructed with an effective slice thickness of 2.5 mm, a field of view of 256 × 256 mm and a matrix size of 512 × 512. For precise anatomical localization, three oil‐filled capsules were placed at the midthigh as anatomical markers (Figure 1B).

### 2.4 Experiment 1: Data and Statistical Analysis

Ultrasound‐derived CSA was measured in triplicate and averaged for analysis in a blind manner. Furthermore, to ensure unbiased measurements, the ultrasound assessments were conducted independently of the MRI analyses. The accuracy of EFOV ultrasound measurements is affected by operator skill (Hernandez‐Belmonte et al. 2022). To assess inter‐rater reliability, two operators independently measured the CSA in a subset of 16 male participants (age: 25.0 ± 1.6 years; height: 1.74 ± 0.04 m; body mass: 63.0 ± 8.2 kg), and the ICC(2, 1) (two‐way random‐effects, absolute agreement, single measures) was calculated. Intra‐rater reliability across the three trials was assessed by calculating the ICC(3, 1) (two‐way mixed‐effects, consistency, single measures) for both male and female participants. The standard error of measurement (SEM) was determined using the formula (SEM = SD $\sqrt{1-\text{ICC}}$, cm^2^) (Van den Broeck et al. 2023). This SEM was then expressed as a coefficient of variation (CV = [SEM/grand mean] × 100). Subsequently, the smallest detectable change (SDC) was derived from the SEM ($\sqrt{2}$ ×SEM×1.96) (Hernandez‐Belmonte et al. 2022). MRI‐derived CSA was determined from the slice corresponding to the oil‐filled markers using specialized analysis software (SliceOmatic, TomoVision, Canada). Pearson's correlation coefficients and Bland–Altman plots were used to evaluate the relationship and agreement between the ultrasound‐ and MRI‐derived CSAs. All statistical analyses were performed using JASP software (version 0.96.0; JASP Team, Amsterdam, Netherlands), with data presented as mean ± SD and significance set at *p* < 0.05.

### 2.5 Experiment 2: Participants

In Experiment 2, twelve participants were initially assigned to an RT group. Data from 11 male participants (age: 21.5 ± 1.0 years; height: 1.71 ± 0.04 m; body mass: 58.9 ± 7.4 kg) were included in the final analysis; one participant was excluded for failing to complete both pre‐ and postassessments due to scheduling conflicts. None of the participants had a history of intense RT. They were all sedentary individuals (Tier 0) who failed to meet the minimum physical activity guidelines (McKay et al. 2022).

### 2.6 Experiment 2: Procedures and Measurement

An 8‐week RT program, targeting the quadriceps femoris via leg press exercises (BM530000; Senoh Corporation, Chiba, Japan), was implemented. The training protocol was adapted from a previous study (Neves et al. 2022). Leg press 1 repetition maximum (1RM) was determined within 3–5 attempts following the standardized ACSM protocol (American College of Sports Medicine 2021) 3–5 days before the start of training. Briefly, after familiarization and a progressive warm‐up, the load was incrementally increased until the participant failed to complete a full repetition through the full range of motion (from full extension to 90° of knee flexion), with 3–5 min of rest between trials. Following the determination of the 1RM, participants performed the RT program 3 days per week with at least 24 hours of recovery between sessions. Each session consisted of three sets at the prescribed intensity, with 2‐min rest intervals between sets. The load was adjusted every three weeks based on repetition maximums: 12RM (weeks 1–3), 10RM (weeks 4–6), and 8RM (weeks 7–8). All sessions were supervised by at least one investigator to ensure proper movement speed and joint positioning.

Ultrasound and MRI assessments were conducted before (pre) and after (post) the 8‐week intervention. Premeasurements were conducted within 1 week before the intervention, and postmeasurements were performed 3–5 days after the final training session to avoid acute effects of RT. Although these data are part of a larger project, none of these data have been reported elsewhere.

### 2.7 Experiment 2: Data and Statistical Analysis

The ultrasound images were analyzed by an investigator who was blinded to participant identity and time point (i.e., pre‐ vs. posttraining). Paired *t*‐tests were performed to evaluate pre‐to‐post changes in muscle CSA for both modalities. Effect sizes were quantified using Cohen's *d*. Bland–Altman plots were constructed to assess the agreement between the changes (Δ) in muscle CSA measured by ultrasound and MRI. Statistical analyses were performed using JASP software (version 0.96.0; JASP Team, Amsterdam, Netherlands), with the significance level set at *p* < 0.05.

## 3 Results

### 3.1 Experiment 1

The ICC between the two operators was excellent [ICC(2,1) > 0.999, 95% confidence interval [CI]: 0.999, 1.000, *p* < 0.001]. The SEM, CV, and SDC were 0.22 cm^2^, 0.31%, and 0.62 cm^2^, respectively. For male participants measured by the operator 1, the ICC for the ultrasound‐derived CSA across the three trials was 0.996 [ICC(3,1) = 0.996, 95% CI: 0.994, 0.997, *p* < 0.001]. The SEM, CV, and SDC were 0.85 cm^2^, 1.19%, and 2.35 cm^2^, respectively. For female participants measured by the operator 2, the ICC for the ultrasound‐derived CSA across the three trials was 0.993 [ICC(3,1) = 0.993, 95% CI: 0.987, 0.997, *p* < 0.001]. The SEM, CV, and SDC were 0.95 cm^2^, 1.73%, and 2.63 cm^2^, respectively. These results indicate that both inter‐rater and intra‐rater reliability of the present ultrasound measurements are regarded as excellent (Koo and Li 2016).

**FIGURE 2:**
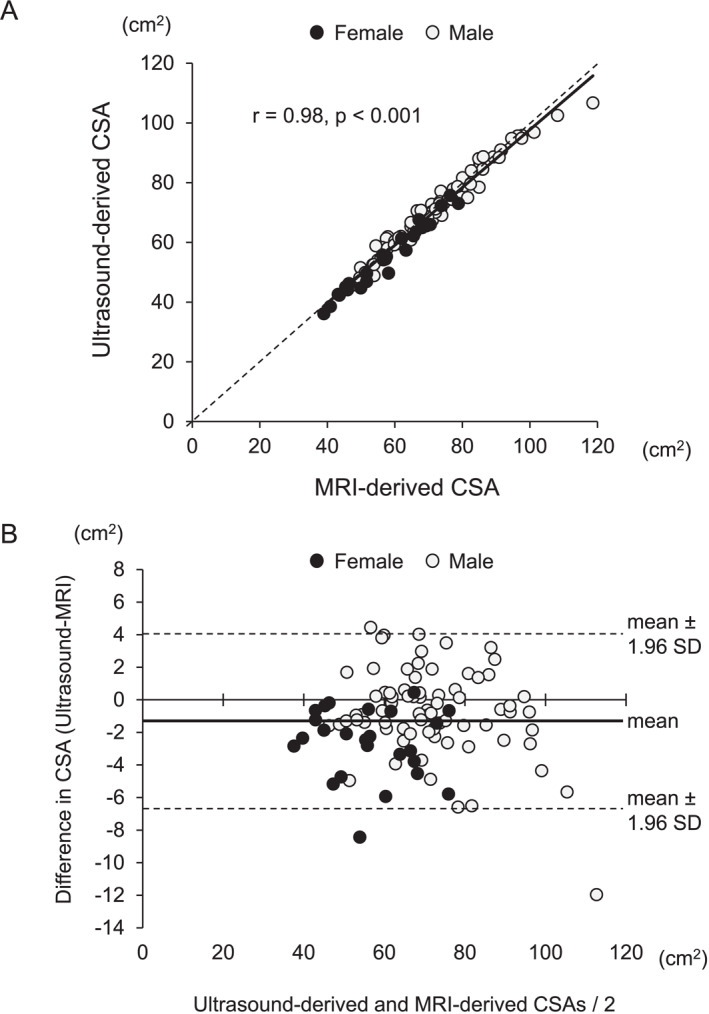
Comparison of quadriceps femoris cross‐sectional area (CSA) measured by magnetic resonance imaging (MRI) and ultrasonography. (A) Scatter plot illustrating the relationship between MRI‐derived and ultrasound‐derived CSA. (B) Bland–Altman plot displaying the agreement between the two imaging modalities. The solid horizontal line represents the mean difference (bias) between the two methods, and the dashed lines indicate the 95% limits of agreement (mean ± 1.96 standard deviation [SD]). Solid black circles show female participants, and light gray circles show male participants.

We observed a very strong positive correlation between ultrasound‐ and MRI‐derived CSAs (*r* = 0.98, *p* < 0.001, Figure 2A). Bland–Altman analysis indicated no significant proportional bias (*r* = −0.10, *p* = 0.32, Figure 2B). However, a small but significant negative fixed bias was present (−1.23 ± 2.70 cm^2^, 95% CI: −1.76, −0.69, *p* < 0.001), indicating a slight underestimation of CSA by ultrasound compared to MRI. Although a few data points fell outside the limits of agreement (upper: 4.06 cm^2^, lower: −6.52 cm^2^), the mean bias was negligible.

### 3.2 Experiment 2

**FIGURE 3:**
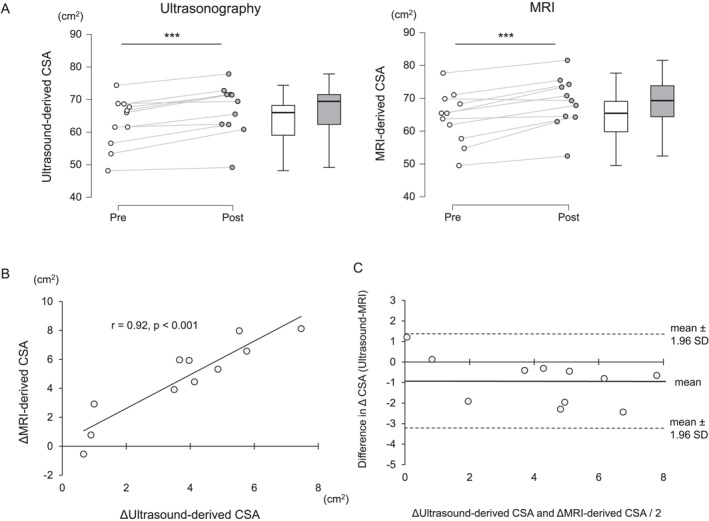
Comparison of longitudinal changes in quadriceps femoris cross‐sectional area (CSA) assessed by ultrasonography and magnetic resonance imaging (MRI). (A) Individual CSA values and boxplots at pre‐ and postintervention assessed by ultrasonography and MRI. (B) Scatter plot illustrating the relationship between the absolute changes (Δ) in ultrasound‐derived and MRI‐derived CSAs. (C) Bland–Altman plot displaying the agreement between the two imaging modalities for the ΔCSA. The solid horizontal line represents the mean difference (bias) in ΔCSA between the two methods, and the dashed lines indicate the 95% limits of agreement (mean ± 1.96 standard deviation [SD]). ***p < 0.001, versus Pre.

Both modalities detected a significant increase in CSA following the 8‐week training period (*p* < 0.001 for both; Cohen's *d* = 1.73 and 1.69 for ultrasound and MRI, respectively; Figure 3A). These results confirmed that the RT program effectively induced hypertrophy of the quadriceps femoris. Furthermore, a strong positive correlation was found between the ΔCSA measured by the two modalities (*r* = 0.92, *p* < 0.001, Figure 3B). Although Bland–Altman analysis revealed no significant proportional bias (*r* = −0.52, *p* = 0.10, Figure 3C), a significant negative fixed bias was present (−0.90 ± 1.13 cm^2^, 95% CI: −1.66, −0.14, *p* = 0.025). Notably, all data points for the training‐induced changes remained within the limits of agreement (upper: 1.32 cm^2^, lower: −3.12 cm^2^).

## 4 Discussion

The primary findings of this study underscore two critical points: (1) the EFOV wireless palm‐sized ultrasound device is a valid modality for assessing quadriceps femoris CSA across a cohort with diverse morphological characteristics; and (2) it possesses sufficient sensitivity to detect muscle hypertrophy induced by RT. These results provide strong empirical support for the practical integration of EFOV wireless palm‐sized imaging systems into both athletic performance monitoring and healthcare applications.

Ultrasonography provides distinct advantages in clinical and research settings, primarily due to its noninvasive nature, cost‐effectiveness, and high safety profile (Franchi et al. 2018). These advantages allow for repeated longitudinal measurements, making it an ideal tool to track changes in both athletes and patients. Previous research using the standard EFOV technique has shown that ultrasound‐derived thigh CSAs are highly comparable to MRI‐derived muscle CSAs (Ahtiainen et al. 2010; Franchi et al. 2020; Hernandez‐Belmonte et al. 2022; Kunimasa et al. 2026; Minnehan et al. 2023; Scott et al. 2017; Stokes et al. 2021; Van den Broeck et al. 2023). However, conventional setups often require complex equipment, which may hinder their use in athletic training fields or certain clinical settings. This study addresses these limitations by utilizing a wireless palm‐sized device equipped with the EFOV technique.

To date, several studies have evaluated the validity of ultrasonography for assessing thigh muscle (rectus femoris, vastus lateralis, vastus medialis, and vastus intermedius) CSA using the EFOV technique (Hernandez‐Belmonte et al. 2022; Kunimasa et al. 2026; Lixandrao et al. 2014; Reeves et al. 2004; Van den Broeck et al. 2023). However, measurement validity has been inconsistent across previous studies. Notably, Noorkoiv and colleagues reported excellent reliability for midthigh (50% of muscle length) quadriceps femoris CSA measurements (Noorkoiv et al. 2010). When comparing EFOV ultrasound with CT, they demonstrated an outstanding ICC of 0.998, along with intra‐ and inter‐rater CVs of 0.6%–0.7%. This extraordinarily high reliability was likely driven by both their use of a custom‐built stabilization device and the inherently large volume of the quadriceps femoris muscle. In the present study, we observed excellent inter‐ and intra‐rater ICCs (> 0.99), acceptable CVs (1.19% for men and 1.73% for women), and a strong positive correlation between the ultrasound‐derived and MRI‐derived measurements (*r* = 0.98). These results are comparable to previous findings using the same device (Matsui et al. 2024). Collectively, the present results demonstrate the validity of this device for assessing thigh muscle CSA against the gold standard, despite the use of a free‐hand technique. Therefore, our findings suggest that the EFOV wireless palm‐sized device provides highly accurate and reliable assessments, extending the scope of prior research by demonstrating that portability does not compromise measurement quality. Specifically, the capability for wireless, compact, and free‐hand measurements, combined with real‐time feedback, underscores the device's utility in space‐limited environments and field‐based athletic settings.

Despite the high correlation, however, we observed a subtle but consistent negative bias (−1.23 cm^2^). These results indicate that ultrasonography slightly underestimates quadriceps femoris CSA compared to MRI. There are several potential physiological and methodological factors that may contribute to this discrepancy. First, the magnitude of the measurement error appears to be dependent on muscle size, and the quadriceps femoris is a particularly large muscle group. A recent study using the EFOV ultrasound technique reported an underestimation that is especially prominent in larger muscles (Kunimasa et al. 2026). The authors suggested that this underestimation could be ascribed to the need to assemble more images using the EFOV method for larger muscles with longer boundaries, which eventually introduces additional errors. This notion may be corroborated by our finding that the participant who exhibited the highest muscle CSA showed the greatest underestimation.

Second, a methodological discrepancy exists regarding the participant's posture during data acquisition. Ultrasound measurements were performed in a seated position, whereas MRI scans were acquired in a supine position. This difference in posture induces gravity‐dependent fluid shifts and alters the hydrostatic pressure within the lower extremities, which can subtly modify muscle architecture and interstitial fluid distribution (Berg et al. 1993). Consequently, these posture‐induced changes in fluid distribution and muscle morphology may explain some of the observed variance, particularly the systematic underestimation of CSA values obtained via ultrasound compared with MRI. Importantly, this underestimation is well‐documented in the literature and represents a known methodological characteristic of ultrasound imaging rather than a device‐specific limitation (Ahtiainen et al. 2010; Kunimasa et al. 2026; Noorkoiv et al. 2010). Given the excellent agreement and strong correlations observed between the two modalities in the present study, this posture‐related discrepancy represents a minor limitation that does not critically compromise the validity or sensitivity of the device.

Third, because muscle boundaries were manually verified and adjusted by the operators, it is plausible that subtle differences in the subjective identification of aponeuroses and fascial borders between the two imaging modalities contributed to this slight underestimation. Finally, measurements were conducted by trained operators with excellent inter‐operator reliability, and no visually identifiable muscle compression was detected. However, the effect of mechanical tissue deformation cannot be entirely excluded. Even minimal probe pressure can compress subcutaneous fat and underlying muscle tissue. Notably, female participants exhibited a slightly greater degree of underestimation (Figure 2), possibly due to sex‐related differences in soft tissue compliance and subcutaneous fat distribution. Nevertheless, we consider that the overall magnitude of underestimation was minimal, suggesting that the impact of sex differences was limited and did not compromise practical utility, particularly when weighed against the portability and efficiency of the ultrasound system. Ultimately, this EFOV wireless palm‐sized ultrasound device serves as a robust and practical tool for evaluating muscle CSA, offering a reliable means of monitoring muscle health and adaptation in diverse environments.

Experiment 2 further demonstrated the longitudinal utility of the EFOV wireless palm‐sized device, as it successfully captured significant muscle hypertrophy following the 8‐week RT intervention. This finding is particularly robust given that the mean hypertrophy (3.77 cm^2^) was greater than the SDC (2.35 cm^2^) established in Experiment 1. Although the ultrasound measurement slightly underestimated the degree of hypertrophy, the difference was minimal (0.90 cm^2^). It is particularly noteworthy that the system exhibited sufficient sensitivity to detect these morphological adaptations, even though the observed increase in ultrasound‐derived muscle CSA was modest (5.98%) due to the short training period. The ability to resolve such modest changes is critically important, as subtle early‐phase adaptations may be obscured by measurement error in less sophisticated field‐based assessments. The inherent advantages of ultrasonography are its noninvasive and nonionizing nature, which permits frequent and repeated longitudinal measurements without the risks associated with radiation exposure. This characteristic allows for the establishment of a more detailed temporal profile of muscle adaptation than would be feasible with higher‐cost, stationary imaging modalities like CT or MRI. Consequently, this EFOV wireless palm‐sized device represents a versatile and robust tool for monitoring dynamic changes in muscle architecture. Beyond the athletic domain, the point‐of‐care capability of this device offers profound implications for clinical settings. Its ease of use at the bedside facilitates the real‐time evaluation of sarcopenia or the progression of various neuromuscular pathologies. By bridging the gap between high‐end laboratory diagnostics and practical clinical application, this technology enables clinicians and practitioners to make data‐driven decisions in both performance optimization and rehabilitative care.

### 4.1 Future Perspectives

Building upon these findings, future research should prioritize validating this EFOV wireless palm‐sized imaging system across broader demographic profiles. Extending validation studies to include other functionally relevant muscle groups would also significantly enhance the utility of this technology for comprehensive musculoskeletal assessments in sports medicine and rehabilitation. In particular, although the quadriceps femoris is important, further investigation is required to determine if similar levels of accuracy and reliability can be achieved in other complex muscle architectures, such as the hamstrings or triceps surae. Furthermore, additional longitudinal studies are necessary to investigate ultrasound‐derived CSA changes in relation to functional outcomes in both athletic training and clinical rehabilitation settings.

## 5 Conclusion

This study validates the use of an EFOV wireless palm‐sized ultrasound device for assessing quadriceps femoris CSA. Our results confirm that measurements from this system are highly correlated with MRI and possess sufficient sensitivity to detect subtle muscle hypertrophy induced by RT. Although a minor negative bias was observed, the magnitude is minimal and falls within a clinically acceptable range. The portability and noninvasive nature of this technology facilitate robust, real‐time assessments at the point of care, representing a significant advancement in the field‐based evaluation of muscular health and adaptation.

## Author Contributions

T.F., Y.H., S.K., M.O., M.S., T.O., and S.A. conceived of the study, carried out the experiment, performed data analysis. T.F., S.K., and S.A. wrote the first draft of the manuscript. Y.H., M.O., M.S., T.O. edited and revised the manuscript. All authors read and approved the final manuscript.

## Funding

This study was partly supported by the Japan Society for the Promotion of Science KAKENHI (Grant Numbers: 23K24750 to S.A.) and the Descente and Ishimoto Memorial Sports Science Foundation (Grant for the year 2023 to S.A.).

## Ethics Statement

This study was approved by the Institutional Review Boards of the University of Electro‐Communications (approval no. 20016) and the Nippon Sport Science University (approval no. 023‐H188). The study conformed to the standards set by the latest revision of the Declaration of Helsinki, except for registration in the database.

## Consent

Informed consent was obtained from all individual participants included in the study.

## Conflicts of Interest

The EFOV wireless palm‐sized ultrasound device used in this study was provided by Furuno Electric Co. Ltd. Furuno Electric Co. Ltd. had no role in the study design, data collection and analysis, interpretation of the results, preparation of the manuscript, or the decision to publish. The authors declare no other competing interests.

## Data Availability

The data that support the findings of this study are available from the corresponding author upon reasonable request.
